# Healthcare Professional Perspectives on Optimizing Patient- and Family-Centered Care in Canadian General Inpatient Pediatrics

**DOI:** 10.3390/jcm15020596

**Published:** 2026-01-12

**Authors:** Karen M. Benzies, Anmol Shahid, Natasha Linda Cholowsky, Deborah McNeil

**Affiliations:** 1 Faculty of Nursing, Cumming School of Medicine, University of Calgary, Calgary, AB T2N 1N4, Canada; dmcneil@ucalgary.ca; 2 Faculty of Nursing, University of Calgary, Calgary, AB T2N 1N4, Canada; anmol.shahid1@ucalgary.ca; 3 Cumming School of Medicine, University of Calgary, Calgary, AB T2N 1N4, Canada; natasha.cholowsky1@ucalgary.ca

**Keywords:** family centered care, family integrated care (FICare), healthcare professional experience, patient and family centered care (PFCC), pediatric nursing, qualitative

## Abstract

**Background/objectives**: Involving parents in the care of hospitalized children can improve outcomes for both patients and families. Our team previously developed a unit-level model of family integrated care that supports families as key members of the neonatal intensive care team. However, the model’s suitability for general inpatient pediatric settings has not yet been explored. To proactively plan for adapting and implementing a feasibility and pilot study of this model in these settings, we examined healthcare professionals’ perspectives on optimizing family integrated care by identifying potential barriers to implementation. **Methods**: We conducted one-on-one semi-structured interviews with ten healthcare professionals along with observational site visits in three general inpatient pediatric units at a large tertiary pediatric hospital in Western Canada. We analyzed data using thematic analysis. **Results**: On average, participants in our study were 35.9 years old, reported 12.2 years of experience in healthcare, were predominantly female, and came from diverse disciplines, and reported substantial healthcare and unit experience. Several themes emerged from the interviews and site observations: resource constraints, workforce challenges, siloed team members, challenges to integrating families in care teams, diverse populations of patients and families, communication barriers, and workflow constraints. Participants indicated these themes may influence integration of families in care in general inpatient pediatric units. **Conclusions**: Our identification of key barriers to integrating families in care offers practical guidance for adapting and implementing family-integrated care in general inpatient pediatric settings.

## 1. Introduction

Over recent decades, pediatric medicine has experienced a paradigm shift toward Patient- and Family-Centered Care (PFCC). This approach emphasizes collaboration among healthcare professionals (HCPs), patients, and families to improve health outcomes and experiences while promoting quality, safety, and effective use of resources [1]. PFCC fosters trust between families and HCPs and can improve both parent and provider experiences. PFCC has been shown to enhance parent satisfaction and confidence, decrease healthcare costs, shorten hospital stays, and reduce readmission rates [2,3,4,5,6,7].

Although PFCC as a philosophy is widely endorsed, its translation into consistent practice remains challenging [8,9,10,11,12,13]. Reported barriers include difficulties clearly defining, operationalizing, and evaluating core PFCC components, limited resources for HCP training, reluctance to modify established routines, and physical or organizational constraints within hospital environments [11,12,13,14,15,16]. Existing PFCC training programs also vary in depth and rigour, with many lacking strong clinical integration or outcome evaluation, highlighting the need for more evidence-informed, practice-oriented education for pediatric HCPs [17,18]. 

In pursuit of standardizing PFCC practice, our team developed a unit-level model of family integrated care (Alberta Family Integrated Care, or FICare) to translate PFCC philosophy into actionable practice. This model integrates parents as active members of the care team from the time of admission by emphasizing relational communication, parent support, and education to foster trust between families and HCPs. Central features include parent participation in bedside rounds, clear negotiation of family–professional roles, and access to psychosocial and peer support networks. The model also generates benefit through standardized multidisciplinary training and implementation tools adaptable to diverse clinical settings [19]. FICare demonstrated improved outcomes for infants, families, and the health system across 14 neonatal intensive care units in Alberta and is now standard practice in these units and in another Canadian province [20,21,22,23,24].

Building on the demonstrated success of FICare in neonatal intensive care units, we began exploring its suitability for general inpatient pediatric settings, where patient and family needs may differ substantially. To date, most PFCC research has focused on pediatric intensive care units [8,25,26,27,28] with relatively limited attention brought to general inpatient pediatric care. We conducted this study to explore HCP perspectives on optimizing PFCC in Canadian general inpatient pediatrics by identifying barriers to future FICare implementation. We anticipated that these findings would inform the adaptation of FICare for general inpatient pediatric units and provide guidance for health system leaders seeking to enhance the quality of care and the experiences of children, families, and healthcare teams in general inpatient pediatric units.

## 2. Materials and Methods

Design: This sub-study represents a two-phase foundational component of a larger implementation science project aimed at determining the feasibility of implementing FICare in general inpatient pediatric units. We designed this work to leverage qualitative data to explore HCP experiences that may influence the practice of family integrated care. A qualitative, interpretive description approach was selected because it aligns with key goals of implementation science, which focuses on understanding factors that help or hinder the uptake of new practices within applied clinical contexts [29,30]. Qualitative methods are well suited to examining the contexts in which implementation occurs, the processes involved, the usefulness of strategies designed to support adoption, and the extent to which planned and actual outcomes align. These methods provide important explanatory insight into both what happens during implementation and why it happens, and are increasingly used to support real-time problem solving and practice improvement [31]. For these reasons, this methodology was chosen to generate meaningful, practice-relevant findings grounded in conceptual and contextual understanding.

Our study was approved by the Conjoint Health Research Ethics Board at the University of Calgary (REB ID: 23-1087). We prepared this manuscript in accordance with the COnsolidated criteria for REporting Qualitative research (COREQ) guidelines [32].

Setting: Between February and June 2024, this sub-study was conducted across three inpatient units at a large academic tertiary pediatric hospital in Western Canada. The hospital provides medical and surgical care in all major pediatric disciplines to 90,000 patients each year.

Participants: Eligible participants included physicians, nurses, allied health professionals, and hospital administrators who had been members of a multidisciplinary team in general inpatient pediatrics for at least one year. There were no exclusion criteria for participants if they met the eligibility criteria. To recruit participants, the Executive Director of Inpatient Care at the hospital sent an email invitation introducing the study. Interested HCPs then contacted our team for further information.

### Data Collection

Semi-structured interviews and surveys: Our team obtained informed consent from participants before scheduling interviews. Using surveys built for the purposes of this study, we collected socio-demographic information from HCPs, including age, gender, education, unit of employment, and length of time working on the unit and in healthcare. Two members of our team with graduate-level backgrounds and 5+ years of experience in patient-oriented research methods conducted semi-structured, one-on-one interviews ranging from 10 to 60 min, completed in person, by phone, or via video conferencing (Zoom; San Jose, CA, USA). A semi-structured interview guide (Supplementary File S1) provided direction for questions and prompts, exploring participants’ roles on the unit, current challenges, and their perspectives on the parental role in caring for hospitalized children. Our team reviewed and verified interview transcripts. We iteratively adapted the interview guide throughout the study to include clarifying questions that supported deeper exploration of emerging concepts.

Observational visits: Our team also conducted observational site visits to assess family integrated care practices on the units, including unit infrastructure, staffing, communication styles, and resources available to families. We transcribed and reviewed observation notes to identify themes and triangulated findings with the interview data during team discussions.

Data Analysis: Using Microsoft Excel (Version 2509, Redmond, WA, USA), our team conducted a thematic analysis of the interviews and observational visit notes following the steps recommended by Braun and Clarke [33]. These steps included: (a) repeated reading to become familiar with the data; (b) generating initial codes; (c) searching for themes; (d) reviewing themes; (e) defining and naming themes; and (f) producing the report. Our team repeatedly read transcripts and identified persistent concepts, noting repetitions in the text, shifts or transitions in content, and similarities and differences across participant responses. We then grouped concepts into domains and developed in-depth descriptions of themes. Data collection and analysis continued until no new concepts emerged, in keeping with recommendations that concepts of saturation guide sample size decisions in qualitative research [34]. Participants did not provide feedback on the findings.

## 3. Results

Ten participants completed one-on-one interviews. On average, participants were 35.9 years old (SD = 8.23). They reported 12.2 years of experience in healthcare (SD = 8.31) and 6.65 years (SD = 7.49) working on their inpatient pediatric unit. Participants included physicians, nursing subspecialties, and allied health professions.

### Emerging Themes

During interviews, several barriers emerged that may influence the implementation of FICare in general inpatient pediatrics. These included: (1) PFCC philosophy in the face of. resource constraint, (2) workforce challenges, (3) siloed multidisciplinary teamwork, (4) difficulties integrating some parents into the care team, (5) patient- and family-level considerations, (6) communication barriers, and (7) workflow constraints.

1. Healthcare professionals believe in PFCC, but are faced with resource constraints

Participants described a strong organizational commitment to PFCC and expressed confidence that pediatric care is already delivered with a high degree of family-centeredness.

“I feel like the leadership and vision of the hospital in general has been very very family centered, focused for a number of years.”

HCP 001

“I feel like our resources are amazing… the funding and resources really show in pediatrics.”

HCP 002

“I see it spark here, and then it kind of fizzles out a little bit there, and then you get another little spark with it.”

HCP 003

At the same time, participants emphasized that resource-constrained environments can make PFCC difficult to prioritize.

“I feel like everyone is kind of trying to do more with less and we see that.”

HCP 001

2. Workforce challenges may come into play

Participants emphasized the effect of staffing shortages on both experienced and less experienced nurses, noting that these pressures shape day-to-day care delivery. At times, care was perceived as great and other times it was perceived less than great. The potential of staffing shortages that lead to burnout was highlighted.

“When you’re newly hired…you’re already stressed out, entering the floor. You’re like, okay, I’ve got my patient assignment. I need to complete all the orders I need to organize my day. You don’t have that like fluidity to your shift quite yet, and I think that’s where we’ll see where we will, where we might see some discomfort with, you know, getting parents involved because they’re just trying to do their part right now and like get comfortable there.”

HCP 004

“Despite a very busy shift with multiple admissions and patients going for surgery, nursing staff on Unit D appeared to work together synchronously, always taking time to interact with families with calmness, a smile, and kind words, even when there was obviously a high demand for their time. Nurses were overheard to say to parents, “What else can I get for you?” There is a calm but effective rhythm to the busyness on Unit D.”

Excerpt from Unit D observational notes

Participants also reported limited time for nursing staff to further develop or refine their PFCC practices:

“I think its going to be the timing. … If you were to add another training, it’s not that it wouldn’t be taken seriously at this exact moment. I think people would get overwhelmed, and it wouldn’t be prioritized potentially.”

HCP 004

Workforce pressures were described as particularly challenging when caring for higher-acuity patients:

“Say, I have a really sick patient and I just have to go there right away, kind of thing, that can be more challenging. I kind of feel like, I don’t know, sometimes I feel like I’m playing catch up the whole shift when that happens and that can be challenging.”

HCP 007

Finally, although systems existed for reporting workflow challenges, participants reported limited time to use them.

“I could write at least one RLS (Reporting and learning System for adverse events) every shift… I just did not even get that far.”

HCP 003

3. Siloing within multidisciplinary teams

Several participants noted that team members often work in parallel rather than collaboratively, creating challenges for care coordination and discharge planning.

“I think it’s everyone’s so focused on their own role.”

HCP 001

“And I think that often you know, like they’re trained to do their job and like this is their scope. And this is what they’re asking about, and they know that they’re so busy. And they’re just trying to, you know, get through everything that they need to get through. But I think that that sometimes means that things are lost between them.”

HCP 005

“Bedside nurse was present during only one instance of rounds. Although she was physically present, the medical team ignored the bedside nurse during rounds.”

Excerpt from Unit C observational notes

4. Difficulties with integrating some parents into the care team

Despite efforts to involve families, participants described situations where engaging parents could be difficult. Competing responsibilities for parents, such as employment, caring for other children, transportation, and financial pressures often limited parents’ ability to be present on the unit.

“Biggest challenges I would say of late, is the children without parents present.”

HCP 001

“A lot of families feel that if by the time they’re on the units, if they miss more work they’ll lose their job. We’ve had a lot of families who have lost their job. So again, you know, that’s not really like that’s reality. And they need to provide for their families. So they need to go to work.”

HCP 006

Participants also noted that parents were sometimes uncertain about which care tasks they were expected to perform, and that nurses varied in their expectations for parental involvement. Participants described needing to provide clear task lists, active coaching, and structured guidance to help parents understand their role.

“It’s just a matter of okay, let’s do this together. We’re gonna do a bed bath together… This is how we do this. Tomorrow you’re on bath duty…When they look at me and they go, “Yeah, now I’m involved. Now I know what to do. These are things I do at home. This is stuff I can do here. You’ve given me a job. Now I have a task I’m not sitting in the corner and like in the corner with my phone, not knowing what to do and fretting over everything. Now I’m involved”

HCP 003

Our team also observed examples of successful parent involvement in care:

“Observed parent/caregiver feeding their child, manipulating bed position, and nurse referenced parent/caregiver having already taken child’s temperature. Parents encouraged to participate in comfort care, distraction, and hygiene. With encouragement and support of the bedside nurse, sometimes parents will record the child’s intake and output.”

Excerpt from Unit C observational notes

5. Patient- and family-level factors that may limit family integration

Participants noted that the diversity within patient and parent populations can influence how effectively families are integrated into the care team. They emphasized that wide variation in patient age and in the acuity or chronicity of illness shapes the level of parental involvement that is feasible in general inpatient pediatric units.

“I’ll be caring for like a baby, a toddler, and a teenager in one shift… it’s always different.”

HCP 007

They also described challenges related to language barriers and previous hospital experiences, both of which may influence how families engage with the care team.

“When you’re on a very busy, heavy unit, it’s easier to just complete your task, then to go get the translator and complete the task in front of the family to offer verbal instruction. So that almost gets skipped a little bit, which I think is part of the problem, too. Just because of the time it’ll take.”

HCP 004

“We sometimes forget this could be their first day in the hospital… and I think the time matters.”

HCP 008

6. Communication dynamics and family integration

Participants described communication as a central factor influencing how families engaged in their child’s care. Four subthemes emerged: respect for parental expertise, inconsistent or delayed information sharing, communication shaped by professional hierarchies, and communication influenced by the variation in bedside rounds. Across these subthemes, participants emphasized that communication breakdowns limited opportunities for PFCC. Research team members also observed several communication-related issues:

“When discussing among team members, often medical terms were not explained. Occasionally, the attending physician asked residents to define terms for families or when patient or parents asked what they meant. In one instance, senior resident asked the junior resident, who was using multiple acronyms, to explain what she was saying so the mother could understand. The mother volunteered that she understood some, but not all, of what the junior resident was saying about her child’s lab work.”

Excerpt from Unit B observational notes

“Team consistently used closed-ended question to ask if the parents had any questions at the end of BSR, e.g., “Do you have any questions.” Opportunity: Use open-ended (circular) questions, “What questions do you have for the team today?”

Excerpt from Unit C observational notes

- Respect for parental expertise

Participants described variation in how parental expertise was acknowledged. Some staff viewed parental insight as essential contextual information that enriched clinical decision-making, while others recognized inconsistencies in how this expertise was valued.

“Can you [mother of child] show me what works best for your kid, and I feel like a lot of us do that because we recognize that, like the parents, are essentially like the team leader, like the kid is the team leader cause.”

HCP 002

“Sometimes a grain of salt, because they don’t always have medical knowledge to back it up, but they often have lots of…insight into their child, and then combined gives us a perspective that we could never even imagine.”

HCP 002

- Inconsistent or delayed information sharing

Participants described frequent challenges ensuring families received timely, accurate, and aligned information about the care plan and discharge. Delayed documentation in the electronic medical record and fragmented communication across team members sometimes resulted in confusion for both staff and families.

“You don’t even know what the changes are… they’re [prescribers] not putting them [orders] in until like 3 h later… it’s chaos sometimes.”

HCP 002

“Messaging not always consistent across specialties… creates extra stress.”

HCP 005

“It’s probably communication within the larger system. So because our patients cross so many different subspecialties we have trouble. And families have said this millions of times to us that everybody’s not always on the same page. So it’s finding ways to get the clinics or sub specialists to talk to each other and align the messaging to the family, because otherwise it’s very confusing.”

HCP 010

- Professional Hierarchies and Trust

Participants reported that communication was often shaped by perceived hierarchy, trust, and individual communication preferences. They highlighted how some of these factors could affect how comfortable families felt raising concerns or asking questions.

“That power imbalance… makes them [parents] feel less able to speak some of their mind.”

HCP 005

“Physicians rule the hospital, you know.”

HCP 005

“Parents… would be more comfortable coming to me than asking questions in front of seven doctors.”

HCP 008

Additional communication challenges included the use of medical jargon:

“Explaining terminology more… not assuming people know what something means.”

HCP 007

7. Workflows affecting integration of families into care teams

Participants described several workflow constraints that shaped bedside rounds, interdisciplinary collaboration, and opportunities for family involvement.

- Unpredictability of bedside rounds timing and structure

Rounding practices were frequently inconsistent, with variable timing, and rotating teams. Participants as well as our team noted considerable variation in how bedside rounds were conducted, sometimes leading to confusion for parents and even staff.

“And then the best answer I can say usually, is, rounds happen between 9 to 12 and ideally be finished before noon, and so the physicians will come by during that time, but we generally don’t elaborate too much.”

HCP 007

“BSR approach varied depending on the attending physician and team members. Sometimes the attending physician will request information about what brought the child to hospital (validating information in the notes); sometimes parents will offer information. Mostly parents listened and responded “no” when asked if they had questions.”

Excerpt from Unit B observational notes

- Limited nursing involvement in bedside rounds

Competing responsibilities and workload pressures often prevented nurses from participating consistently in rounds.

“It’s kind of hard to implement… we have other tasks… it would be interesting to see nursing more involved… I don’t know if it’s possible.”

HCP 007

“There are many reasons why bedside nurses cannot participate in BSR including care for other children on the patient assignment. Staff described how it was challenging for nurses to be present in rounds when they occur in the morning during a busy time for nurses. Orientation for new hires may not include the expectation of participation in BSR for their patients. Additionally, junior nurses may need to build confidence to participate in BSR with the predominately medical team.”

Excerpt from Unit B observational notes

“Half the time in paeds, you don’t even catch rounds. You don’t even know they’ve happened. You don’t know what the changes are, and they’ve made changes, and they not putting them in until like 3 h later, when they have the time. And you don’t know what the plan of care is.”

HCP 002

“Different teams, rounding different times on different units. Sometimes they’ll go back and forth in patients. They’ll have different team members rounding with them. There’s so many more physicians rotating through that it’s harder to get kind of everyone on board”

HCP 002

- Differing parental involvement in bedside rounds

Parents were sometimes absent, uncomfortable, or uncertain about their role in rounds, contributing to missed opportunities for engagement.

“Families aren’t always there for it [bedside rounds], like it’s chaos sometimes”

HCP 002

- Disruptive physical environment

Our team noted that environmental interruptions and noise levels also hindered communication during rounds, affecting family engagement.

“The ambient noise level during BSR was sometimes so high (e.g., jack hammers for construction, music therapy, overhead broadcasts about testing Code Blue system, floor cleaning machines, cleaning/supply carts with noisy wheels) that it was difficult to hear team members’ updates.”

Excerpt from Unit B Unit observational notes

## 4. Discussion

**Figure 1:**
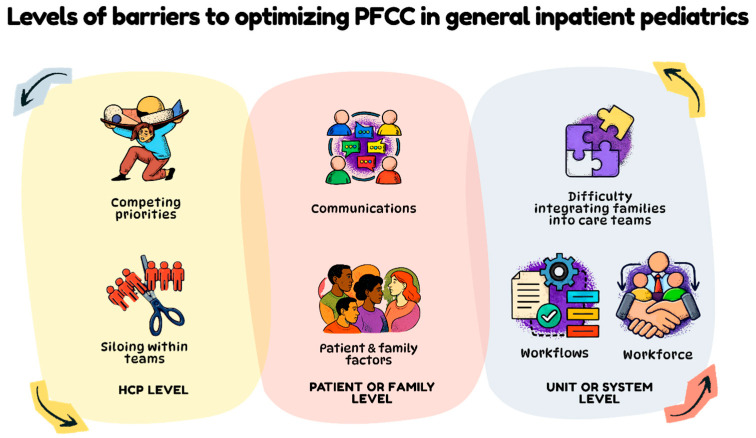
An overview of levels of barriers to optimizing patient and family-centered care in general inpatient pediatrics. Arrows show that barriers and levels of barriers may interact with one another, or compound to create additional challenges to PFCC practice.

This study provides important insights into barriers to implementing PFCC practices in general inpatient pediatrics from the perspective of HCPs. Interviews and observations showed that, despite a clear organizational commitment to PFCC and strong HCP belief in its principles, resource constraints, staffing pressures, and siloed teamwork often limited opportunities to fully integrate parents into care teams. Additional factors such as variability in patient and family needs, communication breakdowns, inconsistent PFCC practices, and unit-level workflow constraints may further limit family integration into care. Together, these challenges may create an ambience of uncertainty around the family’s role and the extent to which parents should be involved in their child’s care, ultimately affecting the translation of PFCC philosophy into practice (Figure 1).

These findings are valuable because they highlight the perspectives of HCPs, whose practical insights are often underrepresented in discussions about PFCC implementation. The findings also provide our team with early direction on potential challenges to the implementation of FICare as we work to adapt the model for use in general inpatient pediatrics. In the sections that follow, we situate our findings within previous work with FICare in other settings, existing literature on barriers and facilitators of PFCC, and opportunities to draw on implementation science and health services optimization research to proactively address these barriers.

Comparing our findings to learnings from FICare’s neonatal application.

Our findings align closely with our team’s observations from the implementation of the FICare model in neonatal intensive care units [35]. They reinforce our expectation that FICare may offer benefits in general inpatient pediatrics, as the model’s core components focus on standardizing PFCC practices through HCP communication training, structured parent support, and ongoing unit-level implementation support [24]. At the same time, the barriers to implementation we identified (e.g., staffing pressures, siloed teamwork, variable family engagement, communication breakdowns) mirror barriers previously noted by our team following implementation of FICare in neonatal intensive care unit [35]. Contextual factors such as competing priorities, limited learning climates, and perceptions of intervention design have influenced FICare uptake across Alberta NICUs and may similarly affect application of the model in general inpatient pediatrics [35].

Comparing previous observations and findings from our team’s work to implement FICare in neonatal settings with our current study is encouraging. The comparison suggests that while FICare has strong empirical support and has garnered interest within general inpatient pediatrics, its adaptation for this setting will require proactively addressing organizational, cultural, and workflow challenges. Insights from this work will guide how we approach these barriers to implementation as we move toward model adaptation and scale-up.

Comparing our findings to the PFCC literature in inpatient pediatrics.

Comparing the findings of our study to the broader pediatric PFCC literature highlights several key similarities and differences. First, much of the existing PFCC research assumes that parents are present and acknowledges their desire to engage in the care of their hospitalized child [36,37]. As a result, the literature is focused on developing guidance on how to engage parents, rather than whether parents are consistently available or able to participate [2]. This contrasts with our findings, where HCPs noted that parents of children with longer hospital stays, or those with competing responsibilities (e.g., caregiving for other children, employment commitments) may be unable to remain at the bedside. Recognizing the extent to which parents can be present, and how much they are realistically able to engage, is essential. Our participants frequently highlighted children without parents present and described how job insecurity, transportation barriers, and other caregiving demands shaped parental availability. These observations suggest that assumptions about parent presence embedded in many PFCC models may need to be reconsidered when applied to general inpatient pediatrics.

Secondly, we observed a notable similarity in the general sense of uncertainty surrounding the family’s role and the considerable variability in how PFCC is enacted. Our participants alluded to this uncertainty as one factor contributing to the unevenness of PFCC practice, which aligns with what has been reported in previous research. Prior literature from global pediatric settings has documented a clear gap between beliefs and practice, noting that although the ethos of PFCC is widely endorsed by HCPs, it is not always implemented consistently in clinical care due to barriers [38,39,40,41]. However, our data indicates an even more diffuse sense of uncertainty. Expectations about what parents should do, and how much staff should coach or rely on them, differed not only across units but also within the same unit. This extends beyond the perception–practice gap identified in previous studies, and we speculate that small, localized differences in day-to-day practice contribute to highly uneven standards of PFCC at the bedside.

Finally, while other pediatric studies note system-level barriers to PFCC, our participants offered more granular descriptions of how unpredictable workflows and bedside rounds shape family integration in general inpatient pediatrics. A scoping review of the literature around family-centered rounds, a very common PFCC intervention in pediatrics, identified logistical constraints such as inconsistent rounding times, staffing pressures, competing priorities, communication breakdowns, and environmental limitations as key barriers to consistent family engagement [42]. Our findings add unit-specific detail, including variable rounding schedules, rotating teams, limited nursing presence on rounds, fluctuating environmental context such as noise and disruptions, diversity in patient and parent population being served, and the absence of virtual tools for communication with parents who are unable to be with their child. Together, these insights highlight setting-specific dynamics that are highly relevant for implementing FICare to general inpatient pediatric units.

Looking ahead—proactively refining an implementation strategy.

Our team aims to leverage implementation science guidance to proactively address the barriers identified in our study as they relate to optimizing PFCC. As we adapt the FICare model for general inpatient pediatrics, we can draw on frameworks such as the Consolidated Framework for Implementation Research [43] as well as real-world examples where these frameworks have been used to plan for success amid contextual challenges similar to those identified in this study, including staffing shortages, workflow disruptions, and uneven practice standards [44]. Structured implementation supports such as team-based problem solving, role clarification, workflow redesign, and protected time for training can improve care delivery and stabilize workforce functioning in complex health systems [45]. We can also rely on insights from our previous large-scale implementation of FICare across all NICUs in Alberta. Reflections from this work, published in an interpretive description study, identified strategic inflection points, including alignment with priorities, user-centered co-design, and embedded governance, that were essential for sustaining complex interventions over time [46]. Leveraging implementation science guidance together with our team’s experience with FICare allows us to embed proactive solutions early and to ensure that communication pathways, staffing structures, and family-integration strategies are intentionally aligned to reduce variability and prevent the workflow bottlenecks identified in this study.

Strengths and limitations: This study adopted a pragmatic approach to qualitative research in implementation science, combining data collection from one-on-one interviews with HCPs and observational site visits. We believe this is a strength of the study, as it fosters a more complete understanding of the practice of PFCC in general inpatient pediatrics by integrating HCP perspectives with real-world, unit-level observations. This approach reflects the methodological flexibility encouraged in implementation science, allowing alignment between the research aims, the nature of the subject matter and context, the techniques used to collect data, and the analytic strategies applied to address the study aims [47].

However, our findings should be considered with several limitations. This study was conducted across multiple units within a single tertiary pediatric hospital, which may limit the transferability of results to other general inpatient pediatric settings. Additionally, we captured the perspectives of HCPs over a relatively short period (five months). As workflows and practices can shift day-to-day based on staffing, patient acuity, and other contextual factors, our site visits represent only a narrow snapshot of PFCC practices. Finally, as in many qualitative studies, sampling bias is possible and individuals with strong views or capacity to participate may have been more likely to enroll. Despite these limitations, we believe the study achieved its goal of identifying key barriers that may influence the implementation of FICare as we move toward pilot testing of the model. Future directions arising from this work include a mixed-methods pilot implementation study of FICare in general inpatient pediatrics, which will use thematic analysis to identify strengths, weaknesses, opportunities, and threats as we advance the model to subsequent stages of research.

## 5. Conclusions

This study identified key barriers that influence the delivery of PFCC in general inpatient pediatric units and highlighted the contextual complexities that may shape the adaptation and implementation of FICare in general inpatient pediatric settings. By drawing attention to factors such as staffing pressures and communication challenges, our findings underscore the need for deliberate, context-sensitive implementation strategies. These insights are expected to inform the proactive adaptation of FICare for general inpatient pediatric settings and contribute to the broader evidence base supporting efforts to strengthen PFCC across pediatric inpatient care.

## Data Availability

The data presented in this study are available on request from the corresponding author. The data are not publicly available to maintain participant privacy.

## Supplementary Materials

The following supporting information can be downloaded at: https://www.mdpi.com/article/10.3390/jcm15020596/s1. Supplementary File S1: The semi-structured interview guide prompts used to collect data around barriers to family integration from healthcare professionals in general inpatient pediatrics.

## Abbreviations

The following abbreviations are used in this manuscript:

FICare	Alberta Family Integrated Care Model
PFCC	Patient- and Family- Centered Care
HCP	Health Care Professionals
