# Supplementary material for: Healthcare Professional Perspectives on Optimizing Patient- and Family-Centered Care in Canadian General Inpatient Pediatrics
Source: J Clin Med. 2026 Jan 12;15(2):596. doi: 10.3390/jcm15020596 (PMC12842568; doi:10.3390/jcm15020596)
Supplement: Supplementary file 1 [file jcm-15-00596-s001.zip › jcm-4046792-supplementary.pdf]

Supplementary File S1: The semi-structured interview guide prompts used to collect data around barriers to family integration from healthcare professionals in general inpatient pediatrics.

Tell me about your experiences as a [*insert nurse, social worker, respiratory therapist, allied healthcare provider*].

1. What does a "**great day**" look like on your unit? (probes: joys, ways you feel you are a valued member of the team, how do you receive positive reinforcement for a "good job")
    a. What works well on your unit (probes: staffing, physical space, parent-staff relationships, policies and procedures, leadership, shift length, shift patterns, ratios)?
2. What are the **biggest challenges** (probes: budget, staffing, space, families, leadership)?
    a. What are the **barriers** that keep you from providing the care that you would like to provide?
    b. What does a "**bad day**" look like on your unit? What makes it difficult?
3. You wear a lot of hats in your role… What do you see as your **main role** on your unit (probes: direct patient/family care, administering medications and feeding, educating parents)?
4. What do you see as your **contributions to the care of families** of children in your unit (probes: parenting education, parent support, challenges, trust)?
    a. What policies and procedures equip you to support parents, and deal with challenging interactions with parents? (if none, what should be in place?)
    b. What would you like your role to be in caring for families/parents?
5. **What role should parents have** in caring for their hospitalized child?
    a. Probes: **how does the parental role change throughout their stay?** fifferences for long vs short stay?
    b. Probes: provide as much care as they are able, how is the physical environment geared to support parents, attending rounds
    c. Probe: you work in an inpatient pediatric setting. As a child grows up, **how does parental involvement in decision-making about their child's care change as the child ages?**
        i. Probe: how do healthcare providers perceive the parents' role in decision-making about their child's care?
    d. *Potential probe:* ***roles of parents in pre- and post-operative care?***
6. What do you see as your **contribution to the multidisciplinary healthcare team**? (Probes: hierarchy)

a. What is your role in contributing to decisions about the child's plan of care?

b. How are decisions about the child's care plan communicated to parents on this unit? (Probes: **trust**, tools available)

c. Can you tell me a bit about **parents' involvement in rounds**

7. If you had an opportunity to change the way care is provided for children and their families on your unit, **what changes would you make?**

   a. What are the '**drivers**' and facilitators for these changes?

   b. What are the **barriers** to making these changes?

   c. Are there any plans in place to implement those changes? If yes, please describe (probes: what is the planned change? What is the timeline? How do you expect the change will improve care in your unit?).

8. What else would you like to tell me about the quality of care provided in your unit?

If we can adapt Alberta FICare for inpatient pediatric units:

1. What are your **thoughts about adopting this model of care** on your unit? (probes: facilitators and challenges)

2. Could this model of care improve outcomes for children and parents on your unit?

   a. If not, what are the greatest challenges?
